# ^123^I‐Metaiodobenzylguanidine Myocardial Scintigraphy in Discriminating Degenerative Parkinsonisms

**DOI:** 10.1002/mdc3.13227

**Published:** 2021-05-07

**Authors:** Mauro Catalan, Franca Dore, Paola Polverino, Claudio Bertolotti, Arianna Sartori, Lucia Antonutti, Alberto Cucca, Giovanni Furlanis, Selene Capitanio, Paolo Manganotti

**Affiliations:** ^1^ Clinical Unit of Neurology, Department of Medical Sciences University Hospital and Health Services of Trieste, University of Trieste Trieste Italy; ^2^ Nuclear Medicine, Imaging Diagnostic Department University Hospital and Health Services of Trieste, University of Trieste Trieste Italy; ^3^ Department of Life Sciences University of Trieste Trieste Italy; ^4^ The Marlene and Paolo Fresco Institute for Parkinson's and Movement Disorders, Department of Neurology NYU School of Medicine New York New York USA; ^5^ Department of Rehabilitation Medicine Villa Margherita Fresco Parkinson Center Vicenza Italy; ^6^ Nuclear Medicine, IRCCS Ospedale Policlinico San Martino Genoa Italy; ^7^ Department of Nuclear Medicine ASST, Grande Ospedale Metropolitano Niguarda Milan Italy

**Keywords:** Parkinson's disease, ^123^I‐MIBG myocardial scintigraphy, atypical parkinsonism, multiple system atrophy, differential diagnosis

## Abstract

**Background:**

^123^I‐Metaiodobenzylguanidine (^123^I‐MIBG) myocardial scintigraphy is a useful technique to differentiate Parkinson's disease (PD) from atypical parkinsonisms, since it is generally abnormal in PD and normal in the latter. Reduction of myocardial MIBG uptake is a supportive feature in the latest PD diagnostic criteria.

**Objectives:**

To explore the clinical contribution of myocardial scintigraphy in discriminating different forms of parkinsonisms, especially when atypical features are present.

**Methods:**

Forty‐one patients with parkinsonism underwent a ^123^I‐MIBG myocardial scintigraphy in our Movement Disorders Center. Disease evolution was reviewed by applying the latest disease criteria for PD, multiple system atrophy (MSA), progressive supranuclear palsy (PSP) and corticobasal syndrome (CBS), as appropriate. Three diagnostic times were defined: T1 (before scintigraphy execution), T2 (immediately after the exam) and T3 (two years later). Early and delayed heart/mediastinum (H/M) ratios and washout rate (WR) were analyzed.

**Results:**

Myocardial scintigraphy showed impaired MIBG uptake in 12 out of 15 patients with a definite PD diagnosis, while normal uptake was found in 20 of 26 patients with no‐PD. Early and delayed H/M ratios were significantly lower in PD compared to overall no‐PD patients and MSA patients. ^123^I‐MIBG myocardial scintigraphy was abnormal in all PD patients with dysautonomia. After ^123^I‐MIBG myocardial scintigraphy (T2), in 9 patients (22%) an improvement of diagnostic accuracy was reached.

**Conclusions:**

Diagnostic accuracy of myocardial scintigraphy in distinguishing PD from atypical parkinsonism was suboptimal. Nevertheless, this study confirmed the relevance of ^123^I‐MIBG myocardial scintigraphy for the discrimination of PD from atypical parkinsonism, especially when dysautonomic symptoms are present.

Parkinson's disease (PD) is a chronic progressive neurodegenerative disorder clinically characterized by bradykinesia, rigidity, tremor at rest, and postural abnormalities with gait impairment.^1^ Atypical parkinsonisms belong to a group of rare degenerative disorders, presenting with a relatively rapidly progressive parkinsonian syndrome, poor response to dopaminergic treatment, and additional clinical signs. Specifically, Multiple system atrophy (MSA) is characterized by a combination of autonomic failure, parkinsonism, and cerebellar signs. Cardinal features of progressive supranuclear palsy (PSP) include postural instability, early falls, and vertical gaze palsy. Finally, corticobasal syndrome (CBS) presents with an asymmetrical parkinsonism typically associated with limb dystonia, myoclonus, apraxia and alien limb phenomena.^2^ Despite their distinct clinical features, the differential diagnosis between PD and atypical parkinsonisms is a recurrent clinical challenge, even among skilled neurologists and movement disorders specialists.^3^ One of the main reasons for this difficulty rests on the slowly progressive nature of these conditions, as well as in the lack of validated, reliable and highly specific biomarkers. In their early stage, atypical parkinsonisms may generally share the same motor phenomenology observed in PD.^4^ As an additional layer of complexity, unusual clinical features (ie, “red flags”), orienting the diagnostic suspicion towards less common forms of parkinsonism, may at times be observed in early PD patients, thus challenging a prompt differential diagnosis.^5^ This has important prognostic implications: atypical parkinsonisms, as compared to PD, are known to be linked to earlier disability, poorer quality of life, and decreased survival. In the past decades, the potential contribution of brain imaging to the differential diagnosis of degenerative parkinsonisms has been increasingly investigated. In this setting, qualitative and quantitative structural magnetic resonance imaging (MRI) represents a useful tool to enhance diagnostic accuracy. Different structural MRI techniques assessing regional changes in tissue volume, signal changes,^6^ and increased deposition of iron,^7^ offer potential surrogate markers of the underlying neurodegenerative process, reflecting neuropathological phenomena such as cell loss, microglial proliferation, and astroglial activation.^8^ Moreover, some nuclear medicine tests have been recognized as potentially helpful tools to discriminate between different forms of parkinsonisms. Among these, ^123^I‐metaiodobenzylguanidine (MIBG) myocardial scintigraphy imaging provides a measure of cardiac sympathetic innervation and it has the potential to differentiate PD from atypical parkinsonisms.^9^ More specifically, in patients with PD and dementia with Lewy bodies (DLB), this nuclear medicine technique is generally abnormal, showing a reduction of tracer concentration in the myocardial muscle.^10^, ^11^, ^12^ Conversely, in patients suffering from atypical parkinsonisms, scans are usually normal.^13^ In light of this evidence, ^123^I‐MIBG myocardial scintigraphy was recently included in the latest PD diagnostic criteria as a supportive feature.^5^ Nevertheless, the precise role of this technique in diagnosing atypical parkinsonisms remains to be ascertained. A large study, conducted on 391 patients with parkinsonism due to different causes, having received MIBG scintigraphy, showed high sensitivity (87.7%) but low specificity (37.7%) of this exam in detecting PD.^14^ Another study confirmed that the discriminatory value of MIBG‐scintigraphy in differentiating MSA from PD is moderate, overall being lower than what attainable by means of diffusion weighted MRI imaging.^15^ Furthermore, a decrease of MIBG uptake was found in approximately 30% of MSA patients, though without any correlation to disease duration or clinical severity.^16^ In spite of these limitations, in recent years, ^123^I‐MIBG myocardial scintigraphy has been increasingly used as a supportive tool in the diagnostic management of parkinsonisms. Over the past decade, our center incorporated this technique in the diagnostic algorithm of patients presenting with a parkinsonian syndrome showing mixed or ambiguous clinical features in the attempt to reach a prompt differential diagnosis. The present study, consisting of a clinical revision of all these cases, aimed to investigate the contribution of ^123^I‐MIBG myocardial scintigraphy in the differential diagnosis of parkinsonisms.

## Methods

### Patients

An observational, retrospective study was conducted on patients with a parkinsonian syndrome referred to the Movement Disorders Unit of the Neurology Department of Trieste, Italy, between 2008 and 2017, who underwent a ^123^I‐MIBG myocardial scintigraphy. The reasons for the execution of the test were the presence of at least one of the following clinical features: suboptimal levodopa response, early falls, autonomic dysfunction at disease onset or rapid progression of symptoms. Patients were screened for medical conditions and medications potentially influencing MIBG scintigraphy results such as diabetes mellitus, various cardiomyopathies, atherosclerotic coronary artery disease, cardiac autonomic neuropathy and pharmacologic compounds including antidepressants, antihypertensive drugs, MAO‐B‐I, Ca2+ channel antagonists, sympathomimetics/sympatholytics. Patients presenting with these confounding medical conditions or incomplete clinical information, were excluded from the study.

The following clinical data were collected for each subject: age at disease onset, clinical phenotype (tremor‐predominant vs. postural instability gait difficult (PIGD), and asymmetrical vs. symmetrical parkinsonism), initial suspected diagnosis, responsiveness to levodopa, levodopa equivalent dose (LED), probable REM behavior disorder (pRBD), presence of clinical features suggesting autonomic dysfunction (urinary/sexual symptoms or orthostatic hypotension, assessed by evaluation of blood pressure and heart rate in supine position and within three minutes of standing, as defined by the Consensus Conference in 1996^17^), and time between disease onset and ^123^I‐MIBG myocardial scintigraphy execution. When available, brain MRI, ^123^I‐Ioflupane SPECT, and autonomic tests were also reviewed. In order to assess the specific contribution of MIBG scan to the diagnostic process, we defined three independent diagnostic time points: time of the clinical evaluation (T1), time of MIBG scan execution (T2), and a 2 years follow‐up assessment (T3). Notably, T1 was defined as the time point in which patients were clinically assessed immediately before undergoing the ^123^I‐MIBG myocardial scintigraphy while T2 was defined immediately after checking ^123^I‐MIBG scintigraphy results. Thus, a very short period of time (no longer than 10 days) intervened between T1 and T2 evaluations. For each diagnostic time point, patients were retrospectively assigned to two mutually exclusive diagnostic categories: PD and no‐PD, the latter including all patients deemed to have MSA, PSP, or CBS. The diagnostic categorization was conducted by two neurologists with expertise on movement disorders (M.A. and L.A.) by applying the latest recommended criteria for PD,^5^ DLB,^18^ MSA,^19^ PSP, and CBS.^20^ In case of two or more different diagnoses, the one with the higher level of certainty was chosen. If the certainty level was found to be the same, patients were defined as “uncertain” on T1 evaluation, then (when possible), categorized on T2 considering also MIBG scintigraphy result. Finally, if patient's clinical features did not satisfy any level of certainty for the specific diagnostic criteria, the case was labeled as “undefined”. Informed consent for data collection was undersigned by all patients included in the present study. The submitted manuscript adheres to the mandates of the Declaration of Helsinki and was approved by our local institution review board.

### ^123^I‐MIBG Myocardial Scintigraphy

For ^123^I‐MIBG scintigraphy, 185 MBq of ^123^I‐MIBG (Mallinckrodt) was injected intravenously. Early and delayed imaging was performed 15 minutes and 4 hours after injection, respectively. The planar imaging was performed with a double–head gamma camera (Siemens Symbya Intevo 2) equipped with a low‐energy, high‐resolution parallel‐hole collimator. The heart to mediastinum ratio (H/M) was calculated by dividing the average counts of the left ventricular ROI by that of the average counts of mediastinum ROI according to the standard method described previously.^9^, ^10^, ^11^ The ^123^I‐MIBG washout rate (WR) background correct (%) was calculated with the following formula: ⦋(early heart counts − early mediastinum counts) − (late heart counts − late mediastinum counts) × 1.21⦌ / early heart counts − early mediastinum counts × 100. According to our database, a delayed H/M ratio of 1.6 or higher and washout 30% or lower are considered normal. If the tracer's uptake was normal or mildly reduced a CT/SPECT was also performed.

### Statistical Analysis

Sensitivity, specificity, positive predictive value (PPV), negative predictive value (NPV), and likelihood ratios of positive and negative results were calculated using published formulas. These parameters were calculated considering only the clinical diagnosis on follow‐up (T3). In order to verify the normal distribution of continuous variables we applied Shapiro–Wilk test. Variables with normal distribution were described as mean ± standard deviation (SD), while non‐normally distributed variables as median (range). Continuous variables were compared between groups using independent samples *T*‐test or Mann–Whitney test, as appropriate. Pearson's chi square test or Fisher's exact test were applied for categorical variables, as appropriate. A two‐tail *P* value <0.05 was considered statistically significant. Statistical analysis was performed by means of IBM Statistical Package for the Social Sciences (SPSS), version 24.0.

## Results

**FIG. 1:**
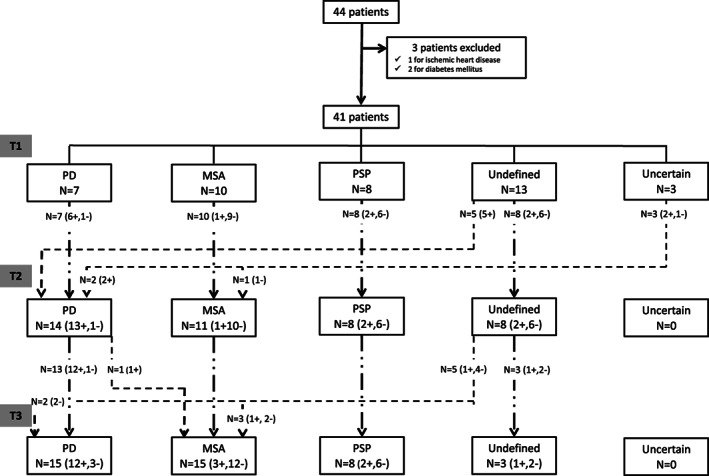
Flow chart resuming the patients' diagnosis at the three different time points (T1, T2, T3) of the study. (+: number of patients with pathological myocardial scintigraphy; −: number of patients with normal myocardial scintigraphy).

Figure 1 shows the study flow chart. As many as 44 patients were deemed eligible. Three of them were excluded from the analysis because of ischemic heart disease (one patient) and diabetes (two patients). Thus, 41 patients were finally included in the study. Brain MRI scans were available for 36 patients and none of them was suggestive of a secondary symptomatic form of parkinsonism. Thirty‐seven patients performed brain ^123^I‐Ioflupane SPECT. In all cases a significant decrement of dopamine transporter (DAT) binding in the striatum was observed. Table 1 highlights demographic and clinical characteristics of the study population, divided into different pathological categories according to the T3 evaluation: 15 patients received a final diagnosis of PD, while 26 patients were considered as no‐PD: in this group 23 received a diagnosis of atypical parkinsonism (15 MSA and 8 PSP, respectively), while 3 patients did not satisfy any clinical criteria and were labeled as undefined. Twenty‐three patients (6 PD, 12 MSA and 5 PSP) presented autonomic dysfunction at T1: upon these 20 patients (4 PD, 11 MSA and 5 PSP) reported urinary or sexual symptoms and 10 patients (4 PD and 6 MSA) had orthostatic hypotension. The incidence of autonomic dysfunction did not differ between PD and no‐PD patients at any time point of the study. Response to levodopa at T1, asymmetrical parkinsonism and ^123^I‐MIBG myocardial scintigraphy, were the only variables which differed significantly between PD and no‐PD patients. Mean values of early and delayed H/M ratio and WR were significantly different in PD compared to overall no‐PD patients (*P* < 0.05) and to MSA patients (*P* < 0.05). When comparing PD to PSP patients, only WR differed significantly (*P* < 0.05), while only a trend towards significance was found for delayed H/M ratio (*P* = 0.065) (Table 2). No significant differences between PSP and MSA were found.

**TABLE 1:** Demographic and clinical characteristics of patients: comparisons between groups according to final diagnosis at T3

Characteristics of patients (n = 41)	PD (n = 15)	No‐PD (n = 26)	MSA (n = 15)	PSP (n = 8)	Undefined (n = 3)	*P value* (PD vs. no PD)
Gender n (%)						
Male	7 (46.7%)	13 (50.0%)	8 (53.3%)	3 (37.5%)	2 (66.7%)	0.837^*^
Female	8 (53.3%)	13 (50.0%)	7 (46.7%)	5 (62.5%)	1 (33.3%)	
Age of onset (y) mean ± SD	63.9 ± 4.6	66.0 ± 7.9	62.8 ± 7.5	70.6 ± 7.2	70.0 ± 4.6	0.368^**^
Age (T1) (y) mean ± SD	68.4 ± 6.5	70.1 ± 8.2	66.1 ± 7.2	75.5 ± 7.5	75.7 ± 1.5	0.493^**^
Disease duration (T1) (y) mean ± SD	3.5 ± 2.8	4.1 ± 3.2	3.3 ± 3.0	4.9 ± 3.4	5.7 ± 4.0	0.658^**^
LED (T1) mean ± SD	490.8 ± 195.5	461.8 ± 262.2	501.7 ± 277.3	410.3 ± 274.0	400.0 ± 173.2	0.720^**^
LED (T3) mean ± SD	636.2 ± 224.7	522.1 ± 251.0	564.5 ± 251.5	525.0 ± 271.2	300.0 ± 0	0.163^**^
MIBG‐SPECT positivity	12(80.0%)	6 (23.1%)	3 (20.0%)	2 (25.0%)	1 (33.3%)	**0.001** ^*^
Clinical features n (%)						
Tremoric parkinsonism (T1)	7 (46.7%)	6 (23.1%)	5 (33.3%)	1 (12.5%)	0 (0%)	0.168^***^
PIGD parkinsonism (T1)	5 (33.3%)	11 (42.3%)	4 (26.7%)	6 (75.0%)	1 (33.3%)	0.742^***^
Asymmetrical parkinsonism (T1)	11 (73.3%)	8 (30.8%)	7 (46.7%)	1 (12.5%)	0 (0%)	**0.011** ^***^
pRBD n (%)	5 (33.3%)	6 (23.1%)	5 (33.3%)	1 (12.5%)	0 (0%)	0.469^***^
Dysautonomia (T1)	6 (40.0%)	17 (65.4%)	12 (80.0%)	5 (62.5%)	0 (0%)	0.191^***^
Dysautonomia (T3)	10 (66.7%)	21 (80.8%)	15 (100%)	6 (75.0%)	0 (0%)	0.453^***^
Urinary symptoms (T1)	4 (26.7%)	16 (61.6%)	11 (73.3%)	5 (62.5%)	0 (0%)	0.052^***^
Urinary symptoms (T3)	8 (53.3%)	21 (80.8%)	15 (100%)	6 (75.0%)	0 (0%)	0.083^***^
Orthostatic hypotension (T1)	4 (26.7%)	6 (23.1%)	6 (40.0%)	0 (0%)	0 (0%)	0.983^***^
Orthostatic hypotension (T3)	5 (33.3%)	10 (38.5%)	10 (66.7%)	0 (0%)	0 (0%)	0.956^***^
Response to levodopa	8 (53.3%)	3 (11.5%)	3 (20.0%)	0 (0%)	0 (0%)	**0.007** ^***^

^*^ Pearson's chi square test.

^**^ Independent samples *T*‐test.

^***^ Fisher's exact test.

PD, Parkinson's disease; No‐PD, total of patients with a diagnosis other than PD; MSA, multiple system atrophy; PSP, progressive supranuclear palsy; Undefined, patients with no established diagnosis; y, years; SD, standard deviation; LED, levodopa equivalent dose; PIGD, postural instability gait difficult; pRBD, probable rem behavior disorder; Bold values differ significantly by comparing the two groups of patients.

**TABLE 2:** MIBG scintigraphy parameters: comparisons between groups

	PD (n = 15)	No‐PD (n = 26)	MSA (n = 15)	PSP (n = 8)	*P‐value* (PD vs. no PD)	*P‐value* (PD vs. MSA)	*P‐value* (PD vs. PSP)	*P‐value* (MSA vs. PSP)
Early H/M ratio*	1.27 (1.12–2.26)	1.66 (1.17–2.26)	1.66 (1.32–2.26)	1.65 (1.17–2.06)	**0.028** ^a^	**0.036** ^a^	0.164^a^	0.810^a^
Delayed H/M ratio*	1.13 (1.02–2.51)	1.64 (1.15–2.44)	1.60 (1.25–2.44)	1.64 (1.15–1.90)	**0.011** ^a^	**0.022** ^a^	0.065^a^	0.656^a^
Wash‐out rate (%)**	56.5 ± 23.1	29.2 ± 13.9	29.9 ± 18.9	28.5 ± 10.5	**<0.001** ^b^	**0.016** ^b^	**0.007** ^b^	0.860^b^

^a^ Mann–Whitney *U* test.

^b^ Independent samples *T*‐test.

* median (range);

** mean ± SD.

PD, Parkinson's disease; No‐PD, total of patients with a diagnosis other than PD; MSA, multiple system atrophy; PSP, progressive supranuclear palsy; Undefined, patients with no established diagnosis; y, years; H/M ratio, heart‐to‐mediastinum ratio; SD, standard deviation; Bold values differ significantly by comparing the two groups of patients.

### Diagnostic Assessment on T1

Immediately before undergoing ^123^I‐MIBG myocardial scintigraphy (T1), 7 patients received a diagnosis of probable PD, 10 patients of MSA (2 probable, 8 possible), and 8 patients of PSP (4 probable, 4 possible). Three patients were considered uncertain, and 13 undefined. Within the latest group, 3 patients had a clinical course suggestive of MSA, but the lack of evidence for autonomic dysfunction did not allow to formally include them in the MSA group at this time point.

### ^123^I‐MIBG Myocardial Scintigraphy Findings on T2

Following ^123^I‐MIBG myocardial scintigraphy, a different diagnosis was obtained in nine patients (22% of all cases). This was more evident for PD: 5 undefined patients on T1 turned into probable PD on T2: in these cases, on T1 evaluation, the number of red flags was not higher than two, but still outweighed the number of supportive features. The finding of cardiac sympathetic denervation on MIBG scintigraphy on T2, increased the number of supportive features, thus counterbalancing the number of red flags. Hence, for these patients, in the absence of any absolute exclusion criteria, the diagnosis changed from undefined to probable PD.^5^ One patient shifted from probable to definite PD: in this specific case, one supportive criterion was yet present at T1 evaluation, without absolute exclusion criteria or red flags; MIBG scan positivity allowed to add the further supportive criteria needed to meet the diagnosis of established PD.^5^ As for the three uncertain patients identified on T1, they received a more defined diagnosis on T2. More specifically, two patients showing a markedly reduced MIBG uptake, were deemed as probable PD, whereas one patient with a normal scan was included in the MSA group. Eight patients remained undefined.

### Final Assessment on T3

On final follow‐up (T3), the number of undefined patients further decreased (n = 3). Three undefined patients were diagnosed with possible MSA as autonomic dysfunction (ie, otherwise unexplained urinary symptoms) became clinically evident. Only 1 patient, who was previously deemed uncertain on T1, then included in the PD group on T2, had his final diagnosis disconfirmed at T3, that is, from probable PD to possible MSA. This occurred because of the emergence of an absolute exclusion criteria, that is, otherwise unexplained cerebellar signs. For all the other patients, the follow‐up evaluation confirmed the diagnosis established on T2 (Fig. 1).

### Diagnostic Match between Scintigraphy Findings and Final Assessment (T3)

When comparing MIBG scintigraphy findings with the final diagnosis established on T3, 80% (12/15) of patients who were assigned to the PD category at T3, showed a significant impairment in the uptake of the radiotracer (sensitivity: 80%; specificity 77%; positive predictive value‐PPV: 66%; negative predictive value‐NPV: 87%). Conversely, the exam resulted normal in 20/26 of subjects who were assigned to the no‐PD category at T3. This group included 12/15 patients with MSA, 6/8 patients with PSP, and 2/3 undefined patients (Table 3). When considering only patients presenting features of ongoing autonomic dysfunction on baseline (T1), myocardial scintigraphy resulted impaired in all (6/6) PD patients and it was normal in 14/17 of no‐PD patients (sensitivity: 100%; specificity: 82%; PPV: 67%; NPV:100%).

**TABLE 3:** Diagnostic match between scintigraphy findings and final assessment (T3)

	PD (n = 15)	MSA (n = 15)	PSP (n = 8)	Undefined (n = 3)
Pathological MIBG uptake	12/15	3/15	2/8	1/3
Normal MIBG uptake	3/15	12/15	6/8	2/3
Sensitivity	80% *	80% **	75% **	67% **
Specificity	76% *	57% **	48% **	45% **

* Test was considered positive in case of pathological MIBG uptake.

** Test was considered positive in case of normal MIBG uptake.

PD, Parkinson's disease; MSA, multiple system atrophy; PSP, progressive supranuclear palsy; Undefined, patients with no established diagnosis.

## Discussion

In this study, we aimed to assess the clinical impact of ^123^I‐MIBG myocardial scintigraphy in the differential diagnosis between PD and atypical parkinsonisms. To this end, we retrospectively analyzed a sample of real‐life patients presenting with a parkinsonian syndrome, in whom a conclusive diagnosis was difficult to obtain. According to a recent meta‐analysis, ^123^I‐MIBG myocardial scintigraphy holds high sensitivity and specificity in differentiating PD from other neurodegenerative parkinsonisms,^21^ despite the studies reported cases of atypical parkinsonisms with reduced MIBG uptake with highly variable incidences.^14^, ^21^, ^22^, ^23^ In agreement with previous studies, we demonstrated that myocardial MIBG uptake is consistently impaired in PD patients as compared to overall no‐PD patients, as well as to MSA and PSP subgroups. In the present study, a cardiac sympathetic denervation was detected in 12/15 PD and 6/26 no‐PD patients, thus resulting in a sensitivity of 80% and a specificity of 77% while discriminating PD from atypical parkinsonisms. Such values are lower compared with those reported in the meta‐analysis.^21^, ^22^ However, it has to be outlined that, in our population, the overall accuracy of ^123^I‐MIBG myocardial scintigraphy may have been underestimated in light of a potential selection bias. As a case in point, only patients with a parkinsonian syndrome showing atypical features were included in the study, while there was a lack of PD patients undergoing a “classical” evolution of the disease. In fact, only 46.7% of PD patients had a tremor predominant parkinsonism, while 33.3% had a PIGD phenotype and only 53.3% of patients on T1 displayed a good response to dopaminergic treatment. Our results are similar to those of a recent study, performed on a sample of patients who presented a rather atypical parkinsonian syndrome without a clinical definite diagnosis. In this study, ^123^I‐MIBG myocardial scintigraphy was associated with 82% sensitivity and 79% specificity in discriminating PD from atypical parkinsonism, but the accuracy raised when MIBG scintigraphy was combined with 123I‐Ioflupane SPECT.^24^

### Contribution of ^123^I‐MIBG Myocardial Scintigraphy to the Diagnostic Process

The main finding of our study is the relevant contribution of MIBG scintigraphy to provide a correct diagnosis in a subset of parkinsonian patients with atypical features and uncertain diagnosis. On this topic, the role of ^123^I‐MIBG scintigraphy in detecting PD, has been previously questioned by some authors in light of its relatively low specificity especially in the early disease stages.^14^, ^25^ Conversely, according to a recent study conducted on a sample of 167 patients with parkinsonian syndrome, ^123^I‐MIBG myocardial scintigraphy was found to have a sensitivity of 94% and a specificity of 65% in detecting early PD patients, with the overall diagnostic accuracy further improving by combining MIBG scintigraphy with a clinical examination including levodopa responsivness.^26^ These findings are supported by a post‐mortem study which demonstrated a sympathetic nerve involvement occurring in 50% of patients who were still in a pre‐clinical phase of the disease.^27^ Furthermore, in PD patients, cardiac sympathetic denervation progresses over time, as suggested by a recent study reporting a decrease in MIBG uptake over a two years follow‐up, especially in patients with normal scans at the beginning of the disease. The study demonstrated that sequential ^123^I‐MIBG scintigraphies may improve the diagnostic ability to discriminate between PD and atypical parkinsonisms.^28^ Our findings are in line with the latter observations, since evidence of cardiac sympathetic denervation was found in 80% PD patients with a relatively brief disease course (mean disease duration of 3.5 ± 2.8 years). In terms of specific diagnostic utility, ^123^I‐MIBG scintigraphy improved the diagnostic accuracy in 22% of our sample. Critically, this percentage raised to 50% (8/16) in patients without a definite diagnosis at baseline (uncertain and undefined groups). Intuitively, these patients are likely to benefit the most from receiving a proper diagnosis, which would have otherwise remained elusive. Interestingly, only one of these patients had a disconfirmation of the diagnosis on the follow‐up evaluation. Finally, one out of the three patients who, despite a normal MIBG uptake, met the diagnosis of PD at the end of the study, deceased in the following year with a final diagnosis of MSA. In this case, a clinical misdiagnosis, due to the relatively brief follow‐up period (2 years), is likely to have occurred.

In the present study, patients with dysautonomia were identified by the presence of orthostatic hypotension, or symptoms suggesting bladder or sexual dysfunction (otherwise unexplained urinary urgency, frequency, incomplete bladder emptying or erectile dysfunction). Only a minority of patients (14) underwent specific autonomic nervous system testing. For this reason, the incidence of autonomic dysfunction in our cohort may have been likely underestimated and this limit is due to the retrospective design of the study. Interestingly, all PD patients with dysautonomia on T1, had an abnormal scan: as a consequence, we found that ^123^I‐MIBG myocardial scintigraphy accuracy in differentiating PD from atypical parkinsonisms improved when only patients with autonomic dysfunction were considered. However, in line with literature,^22^ the present study confirmed that about 20% of MSA patients display an impaired MIBG uptake. According to a recent study, MSA patients have significantly lower values of early and delayed H/M ratios compared to healthy subjects.^29^ Many authors hypothesized that, in the later stages of the disease, a mild peripheral dysfunction, and thus a reduction of MIBG uptake, may occur.^21^, ^22^ This hypothesis is also supported by pathological evidence.^30^

### ^123^I‐MIBG Myocardial Scintigraphy: Which Parameters to Look At?

Finally, specific considerations should be made regarding the different semi‐quantitative measures used to quantify MIBG myocardial uptake (early and delayed H/M and WR). Some authors demonstrated that WR has the highest accuracy in discriminating MSA from PD patients.^23^ WR, is a more recent semi‐quantitative measure obtained from a formula including both early and delayed scans. According to our findings, no differences were detected considering separately early versus delayed H/M ratio, while WR was the only parameter which significantly differed between PD and PSP patients. In our experience, WR may be very useful in border‐line cases, when there is no concordance between early and delayed H/M ratio.

### Limitations

We acknowledge different limitations, mostly inherent to the retrospective nature of this study, including the likelihood of selection biases. In particular the clinical characterization of patients is not extensive (absence of characterization of levodopa response, both in terms of duration and magnitude, lack of categorization of urinary symptoms, absence of data regarding important features like orofacial dyskinesias). Another limitation concerns the disease duration at the time of MIBG scintigraphy execution, which was longer than 3 years, while a prompt differential diagnosis should ideally be established earlier. Furthermore, the relatively small sample size and the lack of a pathological confirmed diagnosis may have further limited the generalizability of our findings. In the effort to partially overcome these limitations, we identified a very short period of time (in the order of a few days) between T1 (time of first clinical assessment) and T2 (time of MIBG scan). This allowed us to consider as virtually negligible other potentially interfering variables on MIBG results between the two evaluations. ^123^I‐MIBG myocardial scintigraphy data were analyzed according to all the available semi‐quantitative parameters, including early H/M ratio, delayed H/M ratio, and WR, thus improving the overall interpretation of our findings. Furthermore, the relatively long follow‐up evaluation (T3) of two years provided us with a reliable observational window on the natural evolution of the disease.

### Conclusions

In the present study, diagnostic accuracy of myocardial scintigraphy in distinguishing PD from atypical parkinsonism was suboptimal. Nevertheless, when evaluating the specific diagnostic contribution of ^123^I‐MIBG myocardial scintigraphy in patients with a parkinsonian syndrome of uncertain nature, this test led to a definite diagnosis in up to 50% of cases. Notably, all the diagnoses established after ^123^I‐MIBG myocardial scintigraphy execution were subsequently confirmed at 2 years follow‐up, except for only one case. Although our study methodology does not allow to draw conclusive recommendations regarding the diagnostic utility of this technique, our results suggest that ^123^I‐MIBG myocardial scintigraphy could be an important tool, especially for the differential diagnosis between PD and MSA in patients reporting dysautonomic complaints. We trust that our data will contribute to the ongoing debate surrounding the future role of ^123^I‐MIBG myocardial scintigraphy in the new diagnostic criteria for MSA. In this setting, large prospective studies, possibly incorporating multimodal biomarkers, are strongly needed.

## Authors' Roles

(1) Research project: A. Conception, B. Organization, C. Execution; (2) Statistical Analysis: A. Design, B. Execution, C. Review and Critique; (3) Manuscript: A. Writing of the first draft, B. Review and Critique.

M.C.: 1A, 1B, 2A, 3A

F.D.: 1A, 1B, 3B

P.P.: 1C, 2B, 3A

C.B.: 1C, 2A, 2B

S.A.: 2A, 2C, 3A

A.L.: 1B, 1C

A.C.: 2C, 3B

F.G.: 1C

S.C.: 1B

P.M.: 1A, 2C, 3C

## Disclosures

**Ethical Compliance Statement:** The study was approved by the local ethics committee “Comitato Etico Unico Regionale – CEUR”. Informed consent for data collection was undersigned by all patients included in the present study. We confirm that we have read the Journal's position on issues involved in ethical publication and affirm that this work is consistent with those guidelines.

**Funding Sources and Conflict of Interest:** No specific funding was received for this work. The authors declare that there are no conflicts of interest relevant to this work.

**Financial Disclosures for the Previous 12 Months:** The authors declare that there are no additional disclosures to report.
